# Clinical efficacy of anti-PD-1 immunotherapy with localized non-ablative irradiation against metastatic lymph node in patients with metastatic gastric cancer

**DOI:** 10.1016/j.ctro.2026.101212

**Published:** 2026-06-02

**Authors:** Kosaku Mimura, Yoshiyuki Suzuki, Shotaro Nakajima, Takashi Ogata, Michael Fehlings, Katsuharu Saito, Yuya Yoshimoto, Daisaku Yoshida, Nozomu Machida, Takanobu Yamada, Hiroyuki Katoh, Hiroyuki Hanayama, Alessandra Nardin, Zenichiro Saze, Fumiaki Takahashi, Takashi Oshima, Koji Kono

**Affiliations:** a Department of Gastrointestinal Tract Surgery, Fukushima Medical University School of Medicine, Fukushima, Japan; b Department of Blood Transfusion and Transplantation Immunology, Fukushima Medical University School of Medicine, Fukushima, Japan; c Department of Radiation Oncology, Fukushima Medical University School of Medicine, Fukushima, Japan; d Department of Multidisciplinary Treatment of Cancer and Regional Medical Support, Fukushima Medical University School of Medicine, Fukushima, Japan; e Department of Gastrointestinal Surgery, Kanagawa Cancer Center, Kanagawa, Japan; f ImmunoScape, Singapore, Singapore; g Advanced Clinical Research Center, Fukushima Global Medical Science Center, Fukushima Medical University School of Medicine, Fukushima, Japan; h Department of Radiation Oncology, Kanagawa Cancer Center, Kanagawa, Japan; i Department of Gastroenterology, Kanagawa Cancer Center, Kanagawa, Japan; j Department of Information Science, Iwate Medical University, Yahaba, Japan

**Keywords:** Radioimmunotherapy, Nivolumab, Gastric cancer, Metastatic lymph node

## Abstract

**Background:**

Cancer immunotherapy combining radiotherapy and anti-PD-1 therapy is a promising treatment strategy. However, the optimal method of radiotherapy to combine with anti-PD-1 therapy has not yet been established. We investigated the clinical efficacy of combinatorial immunotherapy with nivolumab and localized non-ablative irradiation against metastatic lymph node (Nivo-lRT_mLN) in patients with metastatic gastric cancer (m-GC).

**Materials and Methods:**

This exploratory study included 16 patients with m-GC and nivolumab was administered after non-ablative irradiation, 22.5 Gy/5fractions, on one metastatic lymph node measuring 2.0 cm or larger. We evaluated the immunological characteristics associated with the therapeutic efficacy of Nivo-lRT_mLN by multiplex analysis of cytokines, T cell receptor repertoire and flow cytometric analysis using peripheral blood samples.

**Results:**

More than 80% (13 out of 16) of patients had more than 5 metastases and 75% (12 out of 16) of patients had metastases in more than 2 organs. Rate of complete response (CR), partial response (PR), and stable disease (SD) in Nivo-lRT_mLN were 18.7%, 18.7%, and 25.0%. Median survival time was 10.3 months and 4 patients survived for more than five years without recurrence. Before treatment, IL-1ra level was significantly lower and frequency of CD8(+)CD45RO(+)CD27(+)CD127(+) T cells was significantly higher in non-progressors (CR/PR/SD) compared to progressors (PD/NE) and the high group with frequency of CD8(+)CD45RO(+)CD27(+)CD127(+) T cells had significantly better overall survival (OS) than the low group (p = 0.0081). The time-dependent ROC AUC at both the level of IL-1ra and frequency of CD8(+)CD45RO(+)CD27(+)CD127(+) T cells significantly predict OS (p = 0.0193 and p = 0.0442).

**Conclusions:**

These preliminary findings indicated that localized non-ablative irradiation against metastatic lymph node has a potential to enhance the clinical efficacy of anti-PD-1 immunotherapy in patients with m-GC, and IL-1ra level and frequency of CD8(+)CD45RO(+)CD27(+)CD127(+) T cells in peripheral blood before treatment may be candidate biomarkers to predict clinical efficacy of Nivo-lRT_mLN.

## Highlights

• Localized non-ablative irradiation may enhance the efficacy of anti-PD-1 therapy.
• Metastatic lymph nodes may be suitable targets for non-ablative irradiation.
• Serum IL-1ra level may predict overall survival in radioimmunotherapy.
• Central memory CD8 T cells ratio may predict overall survival in radioimmunotherapy.

## Introduction

Immunotherapy with anti-PD-1 mAb is one of the standard treatments in patients with advanced gastric cancer (GC) and it has been established in Asia and Japan that the first line treatment for HER2-negative advanced GC is a combination of nivolumab with chemotherapy [1], [2], [3]. However, median overall survival (OS) and objective response rate (ORR) of combination therapy in first-line therapy ranged from 12.9 to 17.4 months and from 51.3 to 58.0% [2], [3], [4], [5]. Further improvements and biomarker-oriented immunochemotherapy were needed.

Radiotherapy triggers immunogenic tumor cell death (ICD), resulting in activation of anti-tumor immunity through the cancer immunity cycle [6]. We also reported that chemoradiotherapy induced cancer testis antigen-specific cytotoxic T lymphocytes (CTLs) through the ICD in patients with advanced esophageal squamous cell carcinoma and that tumor antigen-specific CTLs were induced after radiotherapy in patients with metastatic GC (m-GC) [7], [8]. Recently, it was also reported that radiotherapy increased the expression of HLA class I on tumor cells, which presented neoantigens derived from DNA damage-induced mutations or tumor neoantigens, and induced CTLs specific for these antigens [9]. These reports indicated that radiotherapy induces tumor antigen-specific CTLs, which are critical for anti-PD-1 immunotherapy, and therefore is considered as one therapeutic strategy to enhance the efficacy of anti-PD-1 immunotherapy. In fact, many clinical trials are ongoing with radiotherapy and anti-PD-1 immunotherapy for various types of malignancies [10], [11], [12].

We conducted the CIRCUIT trial, a Phase I/II study running from March 1, 2018, to August 31, 2021, to evaluate the safety and clinical efficacy of combining anti-PD-1 therapy with radiotherapy in patients with m-GC [10]. At that time, the safety of administering anti-PD-1 therapy and radiotherapy concurrently for GC had not yet been established. Therefore, to ensure patient safety, radiotherapy was administered prior to anti-PD-1 therapy and was restricted to a single lesion that was either the largest or a symptomatic lesion. The protocol design in the CIRCUIT trial was supported by translational rationales. First, we previously reported that cancer antigen-specific CTLs appeared in the peripheral blood approximately two weeks after chemoradiotherapy in patients with advanced esophageal squamous cell carcinoma [7]. Consequently, the CIRCUIT trial protocol initiated anti-PD-1 therapy 15 to 22 days after the start of radiotherapy [10]. Furthermore, in addition to the safety, another reason for targeting a single lesion in radiotherapy was that the primary objective of radiotherapy in the CIRCUIT trial was to activate the cancer-immunity cycle through ICD, leading to abscopal effect [13]. Reports on basic research, including our studies using cancer cell lines, showed that the optimal dose for inducing ICD was approximately 8 Gy x3, rather than an ablative dose [7], [14], [15], [16], [17]. Taking these factors into account, we ultimately adopted a radiotherapy regimen of 22.5 Gy in 5 fractions, which is also applicable to metastases in the radiation-sensitive central nervous system. The CIRCUIT trial revealed that the adding radiotherapy to anti-PD-1 therapy achieved a promising median survival time (MST) in patients with m-GC [10].

Since the CIRCUIT trial enrolled m-GC patients, radiotherapy was administered to target various metastatic sites, including liver metastases, bone metastases, lymph node metastases, primary GCs, and peritoneal disseminated metastases [10]. In all enrolled patients, the disease control rate (DCR) for the non-irradiated lesions was 22.5% and the DCR for the irradiated lesion was 40.0% [10]. In particular, in patients who received radiotherapy to a metastatic lymph node, the DCR for the non-irradiated lesions was 35.7% and the DCR for the irradiated lesion was 57.1%, which were better than those in other metastatic lesions [10]. Furthermore, of the 41 enrollees in the CIRCUIT trial, 4 patients survived for more than five years and 3 of them had irradiated a metastatic lymph node. Irradiation to the metastatic lymph nodes might induce modifications that are effective for anti-PD-1 immunotherapy. Therefore, in this study, we investigated the clinical efficacy of combinatorial immunotherapy with nivolumab and localized non-ablative irradiation against metastatic lymph node (Nivo-lRT_mLN) in patients with m-GC. In addition, we evaluated immunological characteristics that correlate with the clinical efficacy of Nivo-lRT_mLN using peripheral blood samples [8].

## Methods

### Patient cohort and sample collection

This study included 16 patients with m-GC who were treated with Nivo-lRT_mLN (radiotherapy: total 22.5 or 30.0 Gy/5fractions). Localized non-ablative irradiation was performed on one metastatic lymph node measuring 2.0 cm or larger. There were 14 cases from enrollees in the CIRCUIT trial (ClinicalTrials.gov, NCT03453164), and 2 cases, No. 1 and No. 8 cases, were treated with Nivo-lRT_mLN at the Department of Gastrointestinal Surgery, Fukushima Medical University during the CIRCUIT trial period (March 1, 2018–August 31, 2021) [10]. All patients in this study were treated with nivolumab as 3rd line or later chemotherapy because they were treated with nivolumab shortly after nivolumab was first approved for advanced GC in Japan.

To determine the best overall response (BOR) in Nivo-lRT_mLN, we assessed all lesions, including both irradiated lymph nodes and non-irradiated lesions, throughout the entire course of treatment and classified them as complete response (CR), partial response (PR), stable disease (SD), progressive disease (PD) or inevaluable for response (NE) in accordance with the Response Evaluation Criteria in Solid Tumors guideline version 1.1. Patients with PD or NE were classified as progressors and those with CR, PR, or SD were classified as non-progressors in this study. OS was defined as the time from the start date of Nivo-lRT_mLN until the date of death from any cause or the date of the last confirmed survival. Confirmation of survival and collection of the latest clinical information were conducted in May 2026.

Peripheral blood mononuclear cells (PBMC) and plasma samples collected in the CIRCUIT trial were used for immunological analysis. Nivolumab was administered after non-ablative irradiation, 22.5 Gy/5fractions, in the CIRCUIT trial, and samples were collected at 3 time points; before treatment (Pre), after non-ablative irradiation (RT), and before the third administration of nivolumab (Nivo) [8], [10].

### Immunohistochemistry and assessment of staining

Immunohistochemistry was conducted in 14 cases in which biopsy specimens or surgically resected specimens collected prior to Nivo-lRT_mLN were available. Staining for mismatch-repair (MMR) proteins (MLH1, MSH2, MSH6 and PMS2), HLA class I, PD-L1, and PD-L2 was performed as our previous study (Table S1) [18], [19]. Assessment of staining was performed by three independent observers (S.N., K.S., and K.M.), who were blinded to the clinical data. Loss of at least one MMR protein was defined as MMR deficiency (dMMR), and tumors with intact MMR protein expression as MMR proficient (pMMR) [18]. The HLA class I expression on tumor cells was evaluated according to the following criteria: strong, > 30% of tumor cells with dark brown membranous staining; weak, any lesser degree of staining [19]. The PD-L1 and PD-L2 expression value was divided into four groups: > 10% of tumor cells with membranous staining; > 5% of tumor cells with membranous staining; > 1% of tumor cells with membranous staining; negative [19].

### Flow cytometric analysis

We used PBMC samples and the manufacturer’s recommended protocol was used for staining with antibodies to detect each molecule on T cells (Table S2). After compensation was established in the staining set using the fluorochromes, phenotypes of T cell were identified by a classical gating strategy (Fig. S1). The stained cells were measured using a BD FACSCanto II flow cytometer (BD Biosciences, San Jose, CA, USA), and data were analyzed with FlowJo software, version 10.8.1 (Ashland, OR, USA). The previously reported cases were reanalyzed using same gating methods [8].

### Analysis of cytokines in peripheral blood

A multiplex assay was conducted by Fukushima Cell Factory Incorporation (Fukushima, Japan). We measured 24 cytokines in plasma samples in this study, which were reported to be involved in the prognosis of non-small-cell lung cancer patients treated with anti-PD-1 immunotherapy [20]. To measure their concentration, Bio-Plex 200 system (Bio-Rad Laboratories, Hercules, CA, USA) was used with kits from Bio-Rad Laboratories in accordance with the manufacturer’s instructions. We used Bio-Plex Manager Software ver. 6.2 (Bio-Rad Laboratories) to analyze the acquired data.

### Repertoire analysis

Repertoire analysis of TCR β-chain using PBMC samples was conducted as our previous study [8]. In this study, data of seven newly measured cases was added to data of seven previously reported cases and total 14 cases were analyzed [8].

### Statistical analysis

Statistical analyses were performed using R software (version 4.0.3.). Survival analysis was performed using the Kaplan-Meier method and log-rank tests. A two-sided 95% confidence interval of MST was calculated using the Brookmeyer-Crowley method. Wilcoxon's rank-sum test was used to compare two groups. Comparisons between two time points in each target were performed with Wilcoxon's sign rank test. Logistic regression analysis was used to analyze the prediction of tumor response. Cox proportional hazards model regression analysis was used to analyze prediction of overall survival, and time-dependent receiver operating characteristic (ROC) area under the curve (AUC) was calculated using Uno's inverse probability of censoring weighting method. All p-values less than 0.05 were statistically significant, and error bars represent ± standard deviation.

## Results

### Patient’s characteristics

All 16 patients had m-GC and had received at least two chemotherapy regimens prior to enrollment in this study, and more than 80% (13 out of 16) of patients had more than 5 metastases and 75% (12 out of 16) of patients had metastases in more than 2 organs (Table 1). The irradiated lesions for all cases were shown in Table 1. In 16 patients, one case (No.1) exhibited dMMR and two cases (No.1 and No.2) had PD-L1 tumor proportion score more than 10 (Table 1).

**Table 1:** Patients characteristics.

**No.**	**Age group**	**Prior chemotherapy**	**Number of cancer lesions**	**Number of organs with cancer**	**Performance Status**	**Radiotherapy(site, timing, dose)**	**Tumor cells**			
							**MMR**	**PD-L1**	**PD-L2**	**HLA-class1**
**1**	60–69	3	3	2	0	Dorsal pancreatic head, Pre, 22.5 Gy	dMMR	> 10	> 1	weak
**2**	70–79	3	11	2	0	Anterior reigion of common hepatic artery, Pre, 22.5 Gy	pMMR	> 10	> 1	strong
**3**	70–79	3	7	3	0	Porta hepatis, Pre, 22.5 Gy	pMMR	> 1	> 1	strong
**4**	60–69	3	3	1	0	Peri-abdominal aorta, Pre, 22.5 Gy	pMMR	> 1	neg	weak
**5**	70–79	3	9	3	0	Lesser curvature of stomach, Pre, 22.5 Gy	pMMR	> 1	neg	weak
**6**	60–69	3	≧15	1	0	Peri-abdominal aorta, Pre, 22.5 Gy	pMMR	> 1	neg	weak
**7**	60–69	2	7	3	0	Peri-abdominal aorta, Pre, 22.5 Gy	n.d.			
**8**	60–69	3	1	1	0	Dorsal pancreatic head, During, 30.0 Gy	n.d.			
**9**	60–69	2	6	2	0	Lesser curvature of stomach, Pre, 22.5 Gy	pMMR	neg	neg	weak
**10**	70–79	2	11	2	0	Lesser curvature of stomach, Pre, 22.5 Gy	pMMR	> 1	> 1	strong
**11**	80–89	2	9	1	1	Peri-abdominal aorta, Pre, 22.5 Gy	pMMR	> 1	> 1	weak
**12**	50–59	3	≧16	5	0	Peri-abdominal aorta, Pre, 22.5 Gy	pMMR	> 5	neg	weak
**13**	70–79	3	5	2	0	Below the tracheal bifurcation, Pre, 22.5 Gy	pMMR	> 1	neg	strong
**14**	50–59	2	12	3	0	Peri-celiac artery, Pre, 22.5 Gy	pMMR	> 1	neg	weak
**15**	70–79	3	≧11	3	1	Dorsal esophago-jejunal anastomosis, Pre, 22.5 Gy	pMMR	> 5	> 1	strong
**16**	50–59	2	15	4	2	Below the tracheal bifurcation, Pre, 22.5 Gy	pMMR	> 1	neg	weak

Durring, during nivolumab therapy; Pre, before nivolumab therapy; 22.5 Gy, 22.5 Gy/5fractions; 30.0 Gy, 30.0 Gy/5fractions.

### Clinical efficacy

**Fig. 1:**
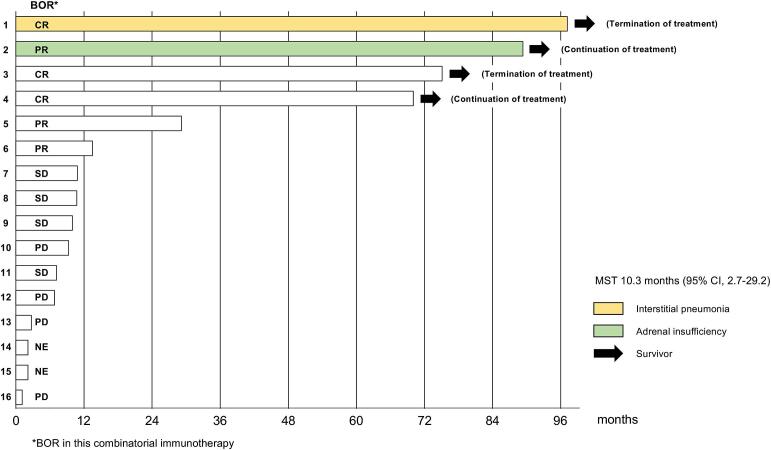
Clinical outcome. Each lane represents a single patient’s data and X axis represents overall survival (OS). Number to the left side of each lane represents identification number of each patient. The best overall response (BOR) in Nivo-lRT_mLN is shown and color of lane shows adverse events that required treatment or discontinuation of nivolumab therapy. CR, complete response; PR, partial response; SD, stable disease; PD, progressive disease; NE, inevaluable for response.

The CR rate was 18.7% (3/16), PR rate was 18.7% (3/16), SD rate was 25.0% (4/16), rate of PD plus NE was 37.5% (6/16) (Fig. 1). Patients diagnosed with PD were promptly transferred to the next line of therapy; in other cases, nivolumab therapy was continued. Chemotherapy other than nivolumab, radiotherapy, and surgery were selected as the next-line treatment. The median follow-up time in this study was 82.2 months (95% confidence interval, 70.0–97.1) and MST was 10.3 months (95% confidence interval, 2.7–29.2), and 4 patients survived for more than five years and were cancer-free (Fig. 1). Patient No.2, who had been diagnosed with PR, was subsequently diagnosed as cancer-free after the residual tumors were surgically resected during treatment. Patient No.1 had dMMR, high PD-L1 expression, and immune-related interstitial pneumonia requiring discontinuation of nivolumab treatment; patient No.2 had high PD-L1 expression and immune-related adrenal insufficiency requiring treatment, both of whom were survivors (Table 1 and Fig. 1). There were only two cases in which patients developed grade 2 or higher adverse events, and adverse events associated with this treatment strategy were comparable to those observed with nivolumab monotherapy [10].

### Immunological parameters associated with BOR

**Fig. 2:**
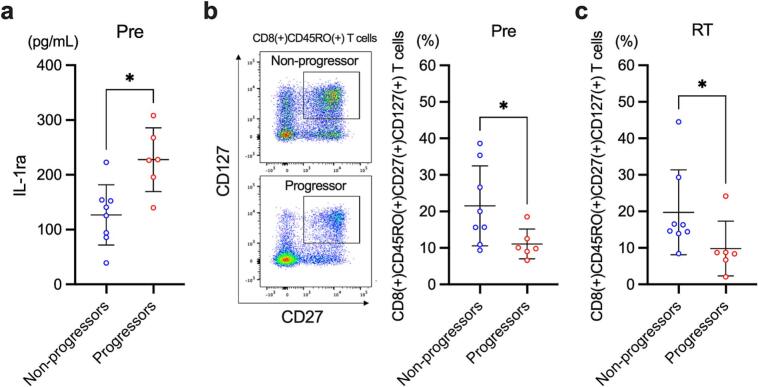
Cytokine and T cell phenotype in peripheral blood in response to treatment. (a) IL-1ra level was analyzed between non-progressors and progressors before treatment (Pre). (b) Representative flow cytometric analysis of CD8(+)CD45RO(+)CD27(+)CD127(+) T cells was shown (left). Frequency of CD8(+)CD45RO(+)CD27(+)CD127(+) T cells was analyzed between non-progressors and progressors at Pre (right). (c) Frequency of CD8(+)CD45RO(+)CD27(+)CD127(+) T cells was analyzed between non-progressors and progressors after non-ablative irradiation (RT). *p < 0.05 and error bars represent ± standard deviation.

We performed multiplex analysis of cytokines using plasma samples, and TCR repertoire and flow cytometric analysis using PBMC samples. We compared each immunological parameter between non-progressors and progressors at each time point (Table S3). At Pre, IL-1ra level was significantly lower and frequency of CD8(+)CD45RO(+)CD27(+)CD127(+) T cells was significantly higher in non-progressors compared to progressors (Fig. 2a and b). Frequency of CD8(+)CD45RO(+)CD27(+)CD127(+) T cells was also significantly higher in non-progressors at RT (Fig. 2c). The frequency of PD-1 or CXCR5 expressing T cells and the diversity or clonality of TCR repertoires were not associated with BOR (Table S3).

**Fig. 3:**
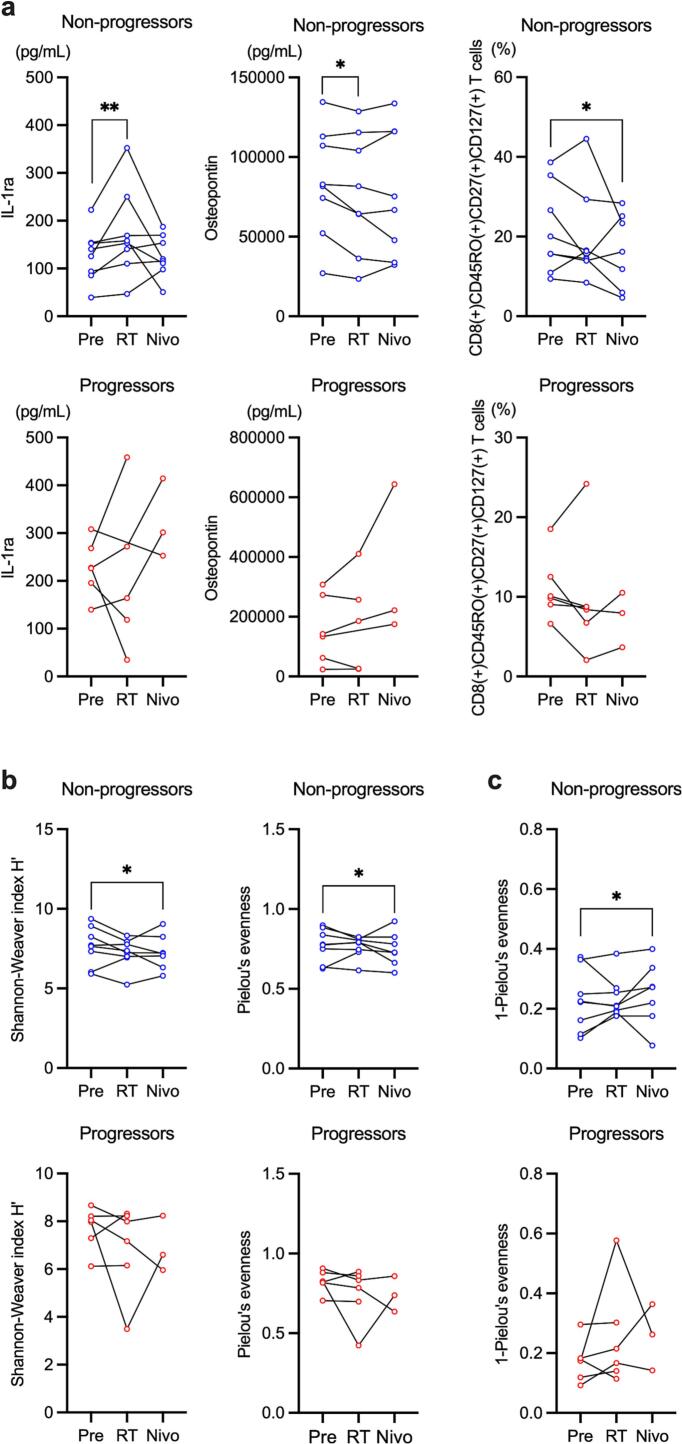
Cytokines, phenotype of T cells, diversity and clonality of T cells in peripheral blood in response to treatment. (a) Modulation of IL-1ra level, osteopontin level, and frequency of CD8(+)CD45RO(+)CD27(+)CD127(+) T cells in response to treatment was evaluated in non-progressors (upper) and progressors (lower). Modulation of diversity (b), Shannon-Weaver index H' and Pielou’s evenness, and clonality (c), 1 – Pielou’s evenness, of T cells in response to treatment was evaluated in non-progressors (upper) and progressors (lower). *p < 0.05, **p < 0.01. Pre, before treatment; RT, after localized non-ablative irradiation; Nivo, before the third administration of nivolumab.

We compared each immunological parameter between Pre, RT, and Nivo (Table S4). IL-1ra level significantly increased and osteopontin level significantly decreased at RT compared to Pre in non-progressors, and the frequency of CD8(+)CD45RO(+)CD27(+)CD127(+) T cells significantly decreased at Nivo compared to Pre in non-progressors (Fig. 3a). Repertoire diversity significantly decreased and repertoire clonality significantly increased at Nivo compared to Pre in non-progressors, and these trends were same as our previous report (Fig. 3b) [8].

### Frequency of CD8(+)CD45RO(+)CD27(+)CD127(+) T cells was involved in OS

**Fig. 4:**
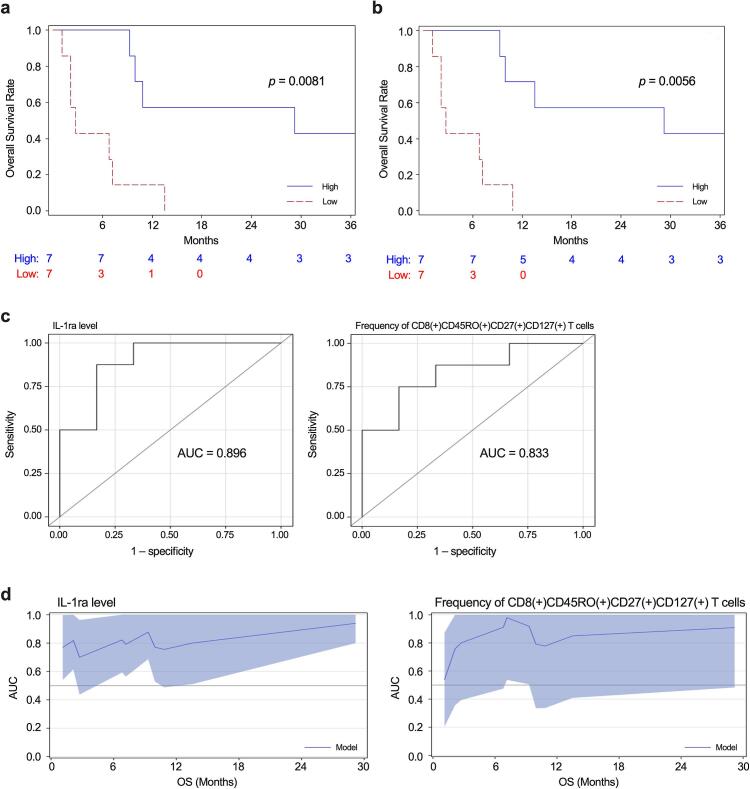
Predicting clinical efficacy using IL-1ra level and frequency of CD8(+)CD45RO(+)CD27(+)CD127(+) T cells. Association between frequency of CD8(+)CD45RO(+)CD27(+)CD127(+) T cells and overall survival (OS) was evaluated. The high and low groups were divided by median value and OS was compared between the two groups before treatment (a) and after non-ablative irradiation (b). The numbers to the right of high and low indicate the number of survivors at each time point. (c) Receiver operating characteristic (ROC) area under the curve (AUC) at IL-1ra level (left) and frequency of CD8(+)CD45RO(+)CD27(+)CD127(+) T cells (right) to predict complete response (CR), partial response (PR), and stable disease (SD) were shown. (d) Time-dependent ROC AUC at IL-1ra level (left) and frequency of CD8(+)CD45RO(+)CD27(+)CD127(+) T cells (right) to predict the risk of OS were shown. Blue color indicated 95% confidence interval. (For interpretation of the references to colour in this figure legend, the reader is referred to the web version of this article.)

We then divided the patients into high and low groups according to the median value of each measured parameter at Pre and RT, and compared them for OS. At both Pre and RT, the high group with frequency of CD8(+)CD45RO(+)CD27(+)CD127(+) T cells had significantly better OS than the low group (p = 0.0081, p = 0.056, respectively) (Fig. 4a and b, Table S5).

### Prediction of clinical efficacy

We evaluated the potential of both IL-1ra level and frequency of CD8(+)CD45RO(+)CD27(+)CD127(+) T cells for predicting clinical efficacy. Regarding the prediction of CR/PR/SD, ROC AUC values at IL-1ra level and frequency of CD8(+)CD45RO(+)CD27(+)CD127(+) T cells were 0.896 and 0.833, respectively (Fig. 4c). Subsequently, we used the time-dependent ROC AUC at the level of IL-1ra and frequency of CD8(+)CD45RO(+)CD27(+)CD127(+) T cells to predict the risk of OS, and both could significantly predict OS (p = 0.0193 and p = 0.0442, respectively) (Fig. 4d).

## Discussion

In the ATTRACTION-2 trial, in which nivolumab monotherapy was administered as the 3rd line treatment for patients with advanced GC, ORR was 11.2% and MST was 5.3 months [21], [22]. We previously reported that combination of non-ablative irradiation with nivolumab for m-GC as the 3rd line setting (CIRCUIT trial) resulted in ORR of 15% and MST of 7.6 months [10]. In this study, we focused on m-GC patients treated with Nivo-lRT_mLN, and ORR was 37.5% (6/16) and MST was 10.3 months (Fig. 1). Furthermore, although the CR rate in the 3-year update of ATTRACTION-2 trial was 0%, CR rate in this study was 18.8% (3/16) [22]. These results suggested that the localized non-ablative irradiation against a metastatic lymph node might enhance the efficacy of nivolumab. In general, the features of responders (CR/PR) to anti-PD-1 immunotherapy in patients with advanced GC have been reported to show immune-related adverse events, dMMR, and high expression of PD-L1, and these features were also observed in some survivors in this study (Table 1 and Fig. 1) [5], [23], [24]. However, 5 out of 6 responders exhibited pMMR in this study, suggesting that Nivo-lRT_mLN may be effective for patients with m-GC exhibiting pMMR as well.

IL-1ra level in peripheral blood plasma was significantly lower in non-progressors compared to progressors at Pre (Fig. 2a). IL-1ra is the IL-1 receptor antagonist that binds to IL-1R1 and inhibits the activity of IL-1α and IL-1β, resulting in attenuation of a diversity of IL-1-related immune and inflammatory responses [25], [26]. Based on previous studies, IL-1 promoted tumor progression through a variety of its biological activities, including immunosuppressive function [25]. On the other hand, however, it was also reported that IL-1α functioned as damage-associated molecular patterns and IL-1α on dendritic cell membrane might play as an adjuvant for tumor neoantigens presentation [25], [26]. Since non-progressors showed significantly lower IL-1ra level in this study (Fig. 2a), there was a possibility that the ICD induced by irradiation might be enhanced through these functions of IL-1, resulting in the activation of cancer-immunity cycle. Furthermore, we indicated that IL-1ra level in peripheral blood had potential in predicting CR/PR/SD and better OS (Fig. 4c left and 4d left). IL-1ra level in peripheral blood at Pre may be a candidate biomarker to predict clinical efficacy of Nivo-lRT_mLN in patients with m-GC.

There are two subsets of memory CD8(+) T cells, central and effector memory CD8(+) T cells, in the peripheral blood. We previously reported that tumor antigen-specific CTLs in peripheral blood of non-progressors tended to be central memory CD8(+) T cells exhibiting CD45RO(+)CD27(+)CD127(+) phenotype in the CIRCUIT trial [8]. In patients with stage IV melanoma, responders to anti-PD-1 immunotherapy had a higher frequency of CD45RO(+)CD62L(+) central memory CD8(+) T cells in peripheral blood before and after treatment than non-responders, and a cluster of circulating central memory T cells expressing CD45RO(+)CD27(+)HLA-DR(+) was expanded in responders after the treatment [27]. Krieg et al. also reported that a subset of circulating central memory CD8(+) T cells exhibiting CD45RO(+)CD27(+)HLA-DR(+) closely resembled a subpopulation of central memory CD8(+) T cells in peripheral blood that was found to expand during anti-PD-1 immunotherapy in stage IV melanoma patients [27], [28]. Based on these reports, the frequency of central memory CD8(+) T cells in peripheral blood may be involved in the efficacy of anti-PD-1 immunotherapy. In this study, at Pre and RT, the frequency of CD45RO(+)CD27(+)CD127(+) central memory CD8(+) T cells in peripheral blood was significantly higher in non-progressors group than in progressors group (Fig. 2b and c), and OS was significantly better in the group with high frequency of circulating CD45RO(+)CD27(+)CD127(+) central memory CD8(+) T cells compared to the group with low frequency (Fig. 4a and b). In addition, we indicated that the frequency of circulating CD45RO(+)CD27(+)CD127(+) central memory CD8(+) T cells might be useful in predicting CR/PR/SD and better OS (Fig. 4c right and 4d right). These results suggested that the frequency of CD45RO(+)CD27(+)CD127(+) central memory CD8(+) T cells in peripheral blood at Pre also may be a candidate biomarker to predict BOR and OS of Nivo-lRT_mLN in patients with m-GC.

Several clinical trials of combinatorial immunotherapy using radiotherapy and anti-PD-1 immunotherapy have been conducted. The outcome of the CIRCUIT trial was favorable and PEMBRO-RT phase 2 randomized clinical trial showed that, in patients with advanced non-small cell lung cancer, the group that received pembrolizumab following stereotactic body radiation therapy (8 Gy x3) had double the ORR compared to the group that received pembrolizumab alone, while adding stereotactic body radiotherapy (9 Gy x3) to nivolumab did not improve the response in patients with metastatic head and neck squamous cell carcinoma [10], [11], [12]. In these clinical trials, the differences in the additive effects of radiotherapy on anti-PD-1 therapy were thought to be influenced by various factors, including the site, dose, timing, and range of radiotherapy, as well as the histological types of malignant tumors such as melanoma, adenocarcinoma, and squamous cell carcinoma. It was recently reported that ablative irradiation and elective nodal irradiation did not induce an immunostimulatory effect and the presence of tumor-draining lymph nodes in the neighborhood of the radiation field was important for enhancing the effect of anti-PD-1 immunotherapy with radiotherapy [6], [9], [29], [30], [31]. The possible explanations for the promising clinical efficacy of Nivo-lRT_mLN were that localized non-ablative irradiation against metastatic lymph node might activate the cancer immune cycle through the migration of antigen-presenting cells loaded with cancer antigens from the irradiated metastatic lymph node to the neighboring tumor-draining lymph nodes [6], [9], [29], [30], [31]. Furthermore, the concurrent combination of radiotherapy and anti-PD-1 therapy might impair CTL function and antigen-presenting machinery in the tumor microenvironment. In this context, Nivo-lRT_mLN might be an effective condition of radiotherapy to enhance the efficacy of anti-PD-1 immunotherapy in patients with m-GC, although it should be carefully considered that the tumor immune microenvironment at the site of radiotherapy may differ depending on the histology of malignant tumors. Further study regarding site, dose, timing, and range of radiotherapy as well as histological types of malignant tumors will be needed to draw solid conclusions.

The limitation of this study is that the total number of enrolled patients was small and the power of statistical analysis was weak. Therefore, the findings of this study are preliminary to clinical application, and further analysis of both the clinical efficacy of this combinatorial immunotherapy and the potential of indicated biomarkers will be desirable in the large-scale clinical trials. In addition, although all patients in this study were treated with nivolumab as 3rd line or later chemotherapy, a combination of nivolumab with chemotherapy is the current first line treatment for HER2-negative advanced GC in Asia and Japan [1], [2], [3]. It is inferred that few patients with advanced GC will be treated with nivolumab monotherapy for the first time in the 3rd line or later chemotherapy. In the future, it is expected that a treatment strategy combining localized non-ablative irradiation to metastatic lymph nodes with current first-line treatments for advanced GC will be necessary for development.

## Conclusion

Although the findings of this study have not yet reached the level of clinical application due to the small sample size, localized non-ablative irradiation, 22.5 Gy/5fractions, against metastatic lymph node has a potential to enhance the clinical efficacy of anti-PD-1 immunotherapy in patients with m-GC, and IL-1ra level and frequency of CD45RO(+)CD27(+)CD127(+) central memory CD8(+) T cells in peripheral blood at Pre may be candidate biomarkers to predict clinical efficacy of Nivo-lRT_mLN.

## Availability of data and material

All relevant data supporting the findings of this study are available within the article and its supplemental material or upon request from the corresponding author.

## CRediT authorship contribution statement

**Kosaku Mimura:** Writing – review & editing, Writing – original draft, Visualization, Validation, Resources, Project administration, Methodology, Investigation, Funding acquisition, Data curation, Conceptualization. **Yoshiyuki Suzuki:** Writing – review & editing, Resources, Investigation. **Shotaro Nakajima:** Writing – review & editing, Visualization, Validation, Investigation, Data curation. **Takashi Ogata:** Writing – review & editing, Resources, Investigation. **Michael Fehlings:** Writing – review & editing, Investigation. **Katsuharu Saito:** Writing – review & editing, Investigation. **Yuya Yoshimoto:** Writing – review & editing, Resources, Investigation. **Daisaku Yoshida:** Writing – review & editing, Resources, Investigation. **Nozomu Machida:** Writing – review & editing, Resources, Investigation. **Takanobu Yamada:** Writing – review & editing, Resources, Investigation. **Hiroyuki Katoh:** Writing – review & editing, Resources, Investigation. **Hiroyuki Hanayama:** Writing – review & editing, Resources, Investigation. **Alessandra Nardin:** Writing – review & editing, Investigation. **Zenichiro Saze:** Writing – review & editing, Resources, Investigation. **Fumiaki Takahashi:** Writing – review & editing, Formal analysis. **Takashi Oshima:** Writing – review & editing, Resources, Investigation. **Koji Kono:** Writing – review & editing, Writing – original draft, Visualization, Supervision, Resources, Project administration, Methodology, Investigation, Data curation, Conceptualization.

## Ethics approval

This study was approved by the Fukushima Medical University Research Ethics Committee (Reference No. 2022–096). This study was conducted in accordance with the 1964 Declaration of Helsinki principles and its later amendments.

## Funding

This work was supported by Japan Society for the Promotion of Science KAKENHI grant number, 23K08114.

## Declaration of competing interest

The authors declare the following financial interests/personal relationships which may be considered as potential competing interests: [Takashi Ogata, Takanobu Yamada, Nozomu Machida and Koji Kono reports speaker fee from ONO Pharmaceutical Co., Ltd. and Bristol-Myers Squibb. The remaining authors have declared no conflict of interest exists.].
