# Supplementary material for: Clinical efficacy of anti-PD-1 immunotherapy with localized non-ablative irradiation against metastatic lymph node in patients with metastatic gastric cancer
Source: Clin Transl Radiat Oncol. 2026 Jun 2;59:101212. doi: 10.1016/j.ctro.2026.101212 (PMC13265895; doi:10.1016/j.ctro.2026.101212)
Supplement: Supplementary Data 1 [file mmc1.pdf]

Figure S1

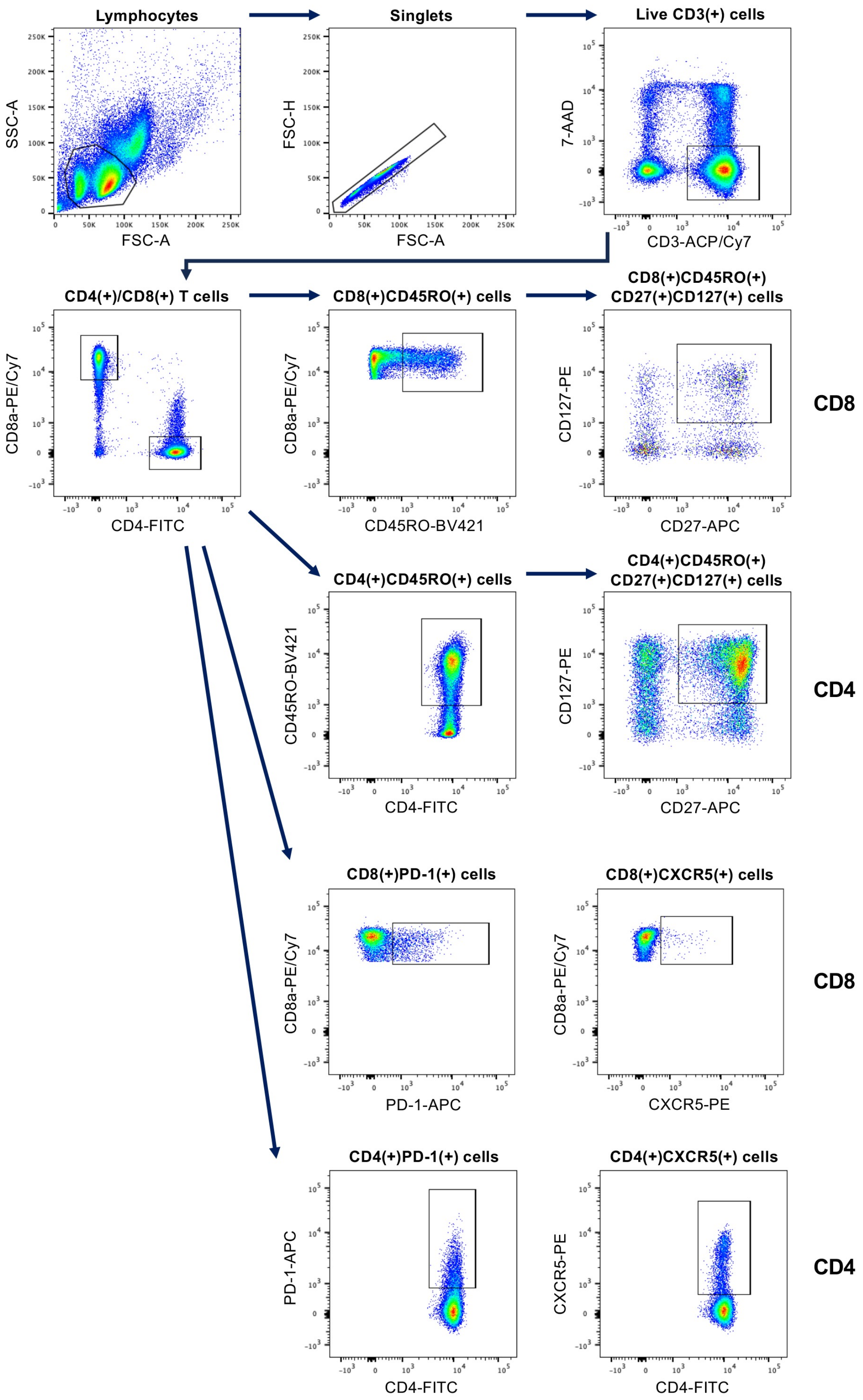

Figure S1 Gating strategy in flow cytometry. For analysis of the central memory T cells, PD-1 or CXCR5 expressing T cells, we first gated the population of lymphocytes in a setting with forward scatter and side scatter, followed by classification of single cells, and then 7-AAD negative CD3-expressing cells were categorized into two groups using CD4 and CD8. Subsequently, we evaluated the frequency of central memory T cells, PD-1 expressing T cells, and CXCR5 expressing T cells. For the central memory T cells, we gated the population to CD45RO expressing cells in CD4 or CD8 positive T cells and measured the frequency of both CD27 and CD127 positive cells. For the PD-1 or CXCR5 expressing T cells, we gated the population to PD-1 or CXCR5 expressing cells in CD4 or CD8 positive T cells and measured their frequency.

Table S1 Antibodies for immunohistochemistry

Antibodies	Company	Catalog number	Souce	Clone	Antigen retrieval	Dilution
MLH1	Agilent / Dako	M3640	Mouse	ES05	pH9 for 10 min at 100℃	1:50
PMS2	Agilent / Dako	M3647	Rabbit	EP51	pH6 for 10 min at 100℃	1:50
MSH2	Agilent / Dako	M3639	Mouse	FE11	pH9 for 10 min at 100℃	1:50
MSH6	Agilent / Dako	M3646	Rabbit	EP49	pH6 for 10 min at 100℃	1:400
PD-L1	Cell Signaling Technology	#13684	Rabbit	E1L3N®	pH9 for 10 min at 105℃	1:400
PD-L2	Cell Signaling Technology	#82723	Rabbit	D7U8C™	pH9 for 10 min at 105℃	1:200
HLA-class1	abcam	ab70328	Mouse	EMR8-5	pH6 for 10 min at 100℃	1:2,000

Table S2 Antibodies for flow cytometric analysis

Product Name	Supplier	Catalog #	Clone
APC/Cyanine7 anti-human CD3 Antibody	BioLegend	300426	UCHT1
FITC anti-human CD4 Antibody	BioLegend	300506	RPA-T4
PE/Cyanine7 anti-human CD8a Antibody	BioLegend	301012	RPA-T8
Brilliant Violet 421™ anti-human CD45RO Antibody	BioLegend	304223	UCHL1
APC anti-human CD27 Antibody	BioLegend	356409	M-T271
PE anti-human CD127 (IL-7Rα) Antibody	BioLegend	351303	A019D5
APC anti-human CD279 (PD-1) Antibody	BioLegend	329907	EH12.2H7
PE anti-human CD185 (CXCR5) Antibody	BioLegend	356903	J252D4
7-AAD Viability Staining Solution	BioLegend	420404	

Table S3 Comparison between non-progressors and progressors at each time point

			p-value		
			Pre	RT	Nivo
Cytokine	APRIL		0.1752	0.4208	0.1846
	BAFF		n.d.	n.d.	0.2616
	sCD163		0.9485	0.8262	n.d.
	CX3CL1		0.5613	0.9417	0.1258
	IL-1ra		0.0169	0.8262	0.0189
	IL-2Rα		n.d.	n.d.	0.0525
	IL-6Rα		0.9485	0.5101	n.d.
	IL-11		> OOR	> OOR	> OOR
	IL-15		n.d.	n.d.	> OOR
	IL-16		n.d.	n.d.	0.475
	IL-17A/F		n.d.	n.d.	0.3354
	IL-21		0.7461	0.4599	1
	IL-23		n.d.	n.d.	0.8364
	IL-25		n.d.	n.d.	> OOR
	IL-33		n.d.	n.d.	0.6057
	CCL5		0.7469	0.7144	n.d.
	CCL13		n.d.	n.d.	0.0525
	CCL20		0.5613	0.3413	n.d.
	CCL23		n.d.	n.d.	0.475
	MMP-1		> OOR	> OOR	n.d.
	Osteocalcin		0.4014	0.1243	0.3583
	Osteopontin		0.2725	0.4208	0.0189
	Pentraxin-3		0.22	0.1243	n.d.
	sTNF-R1		0.4777	0.7144	0.0321
	VEGF-A		n.d.	n.d.	> OOR
Phenotype of T cell	CD4(+)CD45RO(+)CD27(+)CD127(+)		0.6052	0.9485	0.1715
	CD8(+)CD45RO(+)CD27(+)CD127(+)		0.0454	0.0454	0.1715
	CD4(+)PD-1(+)		0.4014	0.1376	0.8197
	CD8(+)PD-1(+)		0.6514	0.2725	0.0682
	CD4(+)CXCR5(+)		0.22	0.7469	0.3619
	CD8(+)CXCR5(+)		0.3329	0.651	0.4941
Repertoire	Diversity	Shannon-Weaver index H'	0.9485	0.8465	0.6485
		Simpson's index 1/λ	0.7469	0.6514	0.8197
		Pielou's evenness	0.4014	0.6514	1
		DE50	0.22	0.4777	1
	Clonality	1-Pielou's evenness	0.4014	0.6514	1

OOR, out-of-range

Table S4 Comparison between two different time points in non-progressors or progressors

					p-value (Wilcoxon signed rank test (median=0))
Cytokine	APRIL		Pre-RT	Non-progressors	0.015625
				Progressors	0.8125
			Pre-Nivo	Non-progressors	0.0546875
				Progressors	0.25
			RT-Nivo	Non-progressors	0.84375
				Progressors	0.5
	sCD163		Pre-RT	Non-progressors	0.640625
				Progressors	0.8125
	CX3CL1		Pre-RT	Non-progressors	0.7421875
				Progressors	1
			Pre-Nivo	Non-progressors	0.7421875
				Progressors	0.25
			RT-Nivo	Non-progressors	0.109375
				Progressors	1
	IL-1ra		Pre-RT	Non-progressors	0.0078125
				Progressors	1
			Pre-Nivo	Non-progressors	1
				Progressors	0.5
			RT-Nivo	Non-progressors	0.3828125
				Progressors	0.5
	IL-6Rα		Pre-RT	Non-progressors	0.3828125
				Progressors	0.1875
	IL-21		Pre-RT	Non-progressors	1
				Progressors	0.625
			Pre-Nivo	Non-progressors	0.7421875
				Progressors	0.75
			RT-Nivo	Non-progressors	0.21875
				Progressors	1
	CCL20		Pre-RT	Non-progressors	0.078125
				Progressors	1
	Osteocalcin		Pre-RT	Non-progressors	0.9453125
				Progressors	0.125
			Pre-Nivo	Non-progressors	0.25
				Progressors	1
			RT-Nivo	Non-progressors	0.109375
				Progressors	0.5
	Osteopontin		Pre-RT	Non-progressors	0.0234375
				Progressors	0.625
			Pre-Nivo	Non-progressors	0.3125
				Progressors	0.25
			RT-Nivo	Non-progressors	0.7421875
				Progressors	0.5
	Pentraxin-3		Pre-RT	Non-progressors	0.7421875
				Progressors	0.4375
	CCL5		Pre-RT	Non-progressors	0.4609375
				Progressors	1
	sTNF-R1		Pre-RT	Non-progressors	0.25
				Progressors	0.625
			Pre-Nivo	Non-progressors	0.640625
				Progressors	0.25
			RT-Nivo	Non-progressors	0.9453125
				Progressors	0.5
Phenotype of T cell	CD4(+)CD45RO(+)CD27(+)CD127(+)		Pre-RT	Non-progressors	0.9453125
				Progressors	0.5625
			Pre-Nivo	Non-progressors	0.21875
				Progressors	0.5
			RT-Nivo	Non-progressors	0.46875
				Progressors	0.25
	CD8(+)CD45RO(+)CD27(+)CD127(+)		Pre-RT	Non-progressors	0.3828125
				Progressors	0.3125
			Pre-Nivo	Non-progressors	0.03125
				Progressors	0.25
			RT-Nivo	Non-progressors	0.375
				Progressors	0.5
	CD4(+)PD-1(+)		Pre-RT	Non-progressors	0.46875
				Progressors	0.03125
			Pre-Nivo	Non-progressors	0.015625
				Progressors	0.25
			RT-Nivo	Non-progressors	0.015625
				Progressors	0.25
	CD8(+)PD-1(+)		Pre-RT	Non-progressors	0.9453125
				Progressors	0.3125
			Pre-Nivo	Non-progressors	0.015625
				Progressors	0.25
			RT-Nivo	Non-progressors	0.015625
				Progressors	0.25
	CD4(+)CXCR5(+)		Pre-RT	Non-progressors	0.640625
				Progressors	1
			Pre-Nivo	Non-progressors	0.6875
				Progressors	0.5
			RT-Nivo	Non-progressors	0.8125
				Progressors	0.25
	CD8(+)CXCR5(+)		Pre-RT	Non-progressors	0.0546875
				Progressors	1
			Pre-Nivo	Non-progressors	0.46875
				Progressors	1
			RT-Nivo	Non-progressors	0.578125
				Progressors	0.75
Repertoire	Diversity	Shannon-Weaver index H'	Pre-RT	Non-progressors	0.109375
				Progressors	0.6875
			Pre-Nivo	Non-progressors	0.046875
				Progressors	0.25
			RT-Nivo	Non-progressors	0.9375
				Progressors	0.75
		Simpson's index 1/λ	Pre-RT	Non-progressors	0.109375
				Progressors	0.84375
			Pre-Nivo	Non-progressors	0.578125
				Progressors	0.25
			RT-Nivo	Non-progressors	0.9375
				Progressors	0.75
		Pielou's evenness	Pre-RT	Non-progressors	0.546875
				Progressors	0.21875
			Pre-Nivo	Non-progressors	0.046875
				Progressors	0.25
			RT-Nivo	Non-progressors	0.21875
				Progressors	0.75
		DE50	Pre-RT	Non-progressors	0.84375
				Progressors	0.21875
			Pre-Nivo	Non-progressors	0.21875
				Progressors	0.25
			RT-Nivo	Non-progressors	0.375
				Progressors	1
	Clonality	1-Pielou's evenness	Pre-RT	Non-progressors	0.546875
				Progressors	0.21875
			Pre-Nivo	Non-progressors	0.046875
				Progressors	0.25
			RT-Nivo	Non-progressors	0.21875
				Progressors	0.75

Table S5 Comparison of overall survival between high and low groups for each measured parameter at the time points of Pre and RT

			p-value	
			Pre	RT
Cytokine	APRIL		0.5643	0.6924
	sCD163		0.5289	0.5931
	CX3CL1		0.4556	0.9386
	IL-1ra		0.0883	0.4818
	IL-6Rα		0.2176	0.897
	IL-11		> OOR	> OOR
	IL-21		0.3964	0.2881
	CCL5		0.38	0.4099
	CCL20		0.8015	0.7285
	MMP-1		> OOR	> OOR
	Osteocalcin		0.7694	0.9827
	Osteopontin		0.0901	0.5099
	Pentraxin-3		0.3451	0.5099
	sTNF-R1		0.2806	0.5931
Phenotype of T cell	CD4(+)CD45RO(+)CD27(+)CD127(+)		0.3078	0.9496
	CD8(+)CD45RO(+)CD27(+)CD127(+)		0.0081	0.0056
	CD4(+)PD-1(+)		0.3951	0.3951
	CD8(+)PD-1(+)		0.7099	0.3525
	CD4(+)CXCR5(+)		0.7644	0.9756
	CD8(+)CXCR5(+)		0.9068	0.7069
Repertoire	Diversity	Shannon-Weaver index H'	0.4383	0.4556
		Simpson's index 1/λ	0.1837	0.1837
		Pielou's evenness	0.2673	0.1837
		DE50	0.2673	0.7589
	Clonality	1-Pielou's evenness	0.2673	0.1837

OOR, out-of-range
